# Hohes Engagement trotz hoher Belastung: arbeitsbezogene Evaluation bei der Umsetzung eines virtuellen, multidisziplinären Extremitätenboards

**DOI:** 10.1007/s00113-025-01588-5

**Published:** 2025-06-12

**Authors:** Joachim Hasebrook, Sibyll Rodde, Marion Laumann, Tobias Hirsch, John Grosser, Steffen B. Roßlenbroich

**Affiliations:** 1 zeb.business school Steinbeis Hochschule, Magdeburg, Deutschland; 2 Universitätsklinikum Münster, Münster, Deutschland; 3 Fachklinik Hornheide, Münster, Deutschland; 4 Fakultät für Gesundheitswissenschaften, Universität Bielefeld, Bielefeld, Deutschland; 5 Klinik für Unfall‑, Hand- und Wiederherstellungschirurgie, Universitätsklinikum Münster, Albert-Schweitzer-Campus 1, Gebäude W1, 48149 Münster, Deutschland

**Keywords:** Traumatologie, Technikakzeptanz, Interprofessionelle Zusammenarbeit, Berufsgruppenvergleich, Versorgungsforschung, Traumatology, Technical readiness, Interprofessional cooperation, Comparison of professional groups, Health services research

## Abstract

**Hintergrund:**

Im Rahmen der Evaluation eines virtuellen, multidisziplinären Experten-Boards zur Verbesserung der Behandlung von Traumata der unteren Extremitäten (Projekt EXPERT) werden neben medizinischen und ökonomischen Outcomes auch arbeitsbezogene Aspekte untersucht. Erste Ergebnisse einer Befragung von Mitarbeitenden, die in EXPERT eingebunden sind, werden hier vorgestellt.

**Material und Methoden:**

Klinikmitarbeitende bearbeiteten einen Online-Fragebogen zu den Themen Arbeitsbelastung, Arbeitsengagement, Technikbereitschaft und arbeitsbezogene Ressourcen. Arbeitsbezogene Ressourcen sind physische, soziale und organisationale Aspekte zur Bewältigung von Arbeitsanforderungen wie kollegiale Unterstützung und Bedeutung der Arbeit. Erfasst wurden Faktoren, die signifikant auf Arbeitsengagement und Technikbereitschaft einwirken.

**Ergebnisse:**

Zu Beginn des Projekts war das Arbeitsengagement sehr hoch, obwohl die individuelle Arbeitsbelastung ebenfalls hoch war. Bei hoher individueller Belastung stieg die Bereitschaft, Technik zu nutzen, wenn ausreichend arbeitsbezogene Ressourcen verfügbar waren. Nichtärztliches Personal wies insbesondere bei geringen Ressourcen eine höhere Technikbereitschaft auf. Bei hoher teambezogener Belastung, z. B. durch Konflikte, und nur wenigen Ressourcen war die Technikbereitschaft besonders gering.

**Diskussion:**

Vielfältige arbeitsbezogene Ressourcen stärken nicht nur das Arbeitsengagement, sondern auch die Akzeptanz digitaler Innovationen. Bei hoher individueller Beanspruchung nehmen Mitarbeitende, insbesondere Angehörige der nichtärztlichen Berufe, Technikeinsatz als hilfreich wahr. Voraussetzung dafür ist, dass die Zusammenarbeit im Team funktioniert.

**Graphic abstract:**

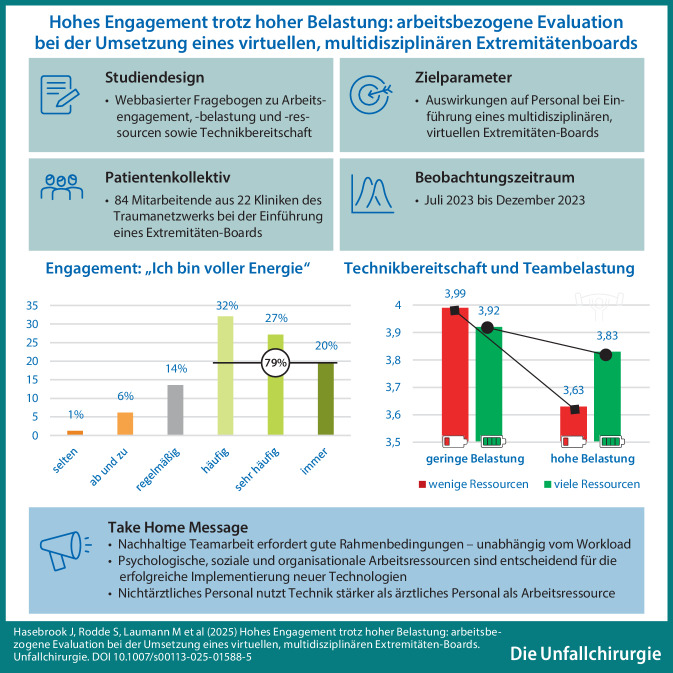

## Abstract (en)

**Background:**

As part of the evaluation of a virtual multidisciplinary expert board to improve the treatment of trauma to the lower extremities (project EXPERT), work-related aspects are examined in addition to medical and economic outcomes. Initial results of a survey of employees involved in EXPERT are presented here.

**Material and methods:**

Clinical staff worked on an online questionnaire on the topics of workload, work commitment, work-related resources and technical readiness. Work-related resources are physical, social and organizational aspects for overcoming work demands, such as peer support, feedback, opportunities to learn and meaningfulness of work. Factors that have a significant effect on work commitment and willingness to work were recorded.

**Results:**

At the beginning of the project, the work commitment was very high, although the individual workload was also high. When individual stress was high, the willingness to use technology increased if sufficient work-related resources were available. Nonmedical staff showed a high willingness to use technology, especially with limited resources. With high team-related stress, e.g. due to conflicts and only a few resources, the willingness to use technology was particularly low.

**Discussion:**

Diverse work-related resources not only strengthen work commitment but also the acceptance of digital innovations. In the case of high individual stress, employees, especially members of the nonmedical professions, perceived the use of technology as helpful. The prerequisite for this is that the cooperation in the team works.

## Einleitung

### Teilnehmende Kliniken

Teilnehmende Kliniken sind: Diakonieklinikum Rotenburg, Marienhospital Osnabrück, Klinik am Hellweg GmbH Bad Sassendorf, St. Marien-Hospital Hamm GmbH, Evangelisches Krankenhaus Oldenburg, Ludmillenstift Meppen, Klinikum Bremen-Mitte, Klinikum Vest, Klinikum Gütersloh, Klinik Münsterland Bad Rothenfelde, Johannes Wesling Klinikum Minden, Fachklinik Bad Bentheim, Klinikum Ibbenbüren, St. Marien-Krankenhaus Ahaus, Euregio-Klinik Nordhorn, Josephs-Hospital Warendorf, Christophorus-Kliniken Coesfeld, Diakonie Klinikum Jung-Stilling Siegen, Evangelisches Krankenhaus Mülheim, Klinikum Osnabrück GmbH, Stiftungsklinikum PROSELIS Recklinghausen, St. Marien Hospital Lünen, Maria-Josef-Hospital Greven, Christliches Krankenhaus Quakenbrück, St. Antonius-Hospital Gronau, Clemenshospital Münster, Herz-Jesu-Krankenhaus Münster-Hiltrup, UKM Steinfurt, und Evangelisches Klinikum Bethel Bielefeld, Fachklinik Hornheide, Universitätsklinikum Münster. Weitere Informationen zum Projekt im Internet unter https://expert-projekt.de/home.

### Hintergrund: Evaluation eines virtuellen, multidisziplinären Extremitäten-Boards (EXPERT)

In Deutschlands Schockräumen wird interdisziplinär nach konsentierten diagnostischen und therapeutischen Algorithmen gehandelt. In der subakuten Traumatologie fehlen jedoch multiprofessionelle Behandlungsstrukturen, die angesichts der zunehmenden Spezialisierung und des demografischen Wandels immer wichtiger werden [26]. Betroffen sind v. a. Patienten mit offenen Frakturen bei Indikation einer plastischen Deckung, oder Patienten, die fraktur- bzw. implantatassoziierte Infektionen aufweisen. Internationale Studien belegen bei dieser Patientenklientel bessere Heilungserfolge bei interdisziplinärer klinischer Zusammenarbeit [10, 13, 21] und der Anwendung gemeinsamer Standards in Diagnostik und Therapie [16, 17, 19, 26]. Zudem können hohe Behandlungskosten [8, 15, 25], die aufgrund von Verlegungen, redundanter Diagnostik, erneuter Operationen und langen Krankschreibungen entstehen [9, 12, 20], erfolgreich reduziert werden [10, 13, 17, 19].

Bisher gibt es in Deutschland nur an ausgewählten Zentren interdisziplinäre Behandlungsansätze für traumabezogene, plastische oder infektiöse Fragestellungen. Hier setzt das vom Innovationsfonds des gemeinsames Bundesausschusses (G-BA) geförderte Projekt EXPERT (Extremitäten-Boards zu Prozessoptimierung, Evaluation, Risikominimierung und Therapieoptimierung bei Frakturen mit Weichteilschäden oder postoperativer Infektion der unteren Extremitäten im Traumanetzwerk) an. Durch EXPERT sollen im Gesundheitssystem sowohl eine Zusammenarbeit zwischen chirurgischen und nichtchirurgischen Disziplinen als auch eine zunehmende Standardisierung von Diagnostik und Therapie erprobt werden.

Die Innovation von EXPERT besteht darin, dass Ärzte aus Krankenhäusern der Region über eine telemedizinische Plattform einer interdisziplinären Expertengruppe ihre Behandlungsfälle vorstellen. Die Expertengruppe aus dem Umfeld des Universitätsklinikums Münster verfügt über Expertise in plastischer Chirurgie, Pharmazie, Infektiologie, Gefäßchirurgie, Hygiene, Angiologie, Radiologie, Mikrobiologie und Unfallchirurgie. Dieses multiprofessionelle Experten-Board gibt zeitnah eine gemeinsame Behandlungsempfehlung für den eingereichten Behandlungsfall ab [22].

Ziel der neuen Versorgungsform ist die Reduktion von Komplikationen während der Behandlung. Zudem sollen Verringerungen der Behandlungsdauer und der Komplikationsrate sowie der Behandlungskosten erreicht werden. Zur empirischen Evaluation von EXPERT werden neben patientenbezogenen klinischen Erfolgskriterien und ökonomischen Outcomes auch arbeitsbezogene Aspekte erfasst.

#### Arbeitsbezogene Evaluation von EXPERT

Für die arbeitsbezogene Evaluation des EXPERT-Projekts wird das Job-Demands-Resources(JDR)-Modell [6, 7] herangezogen. Das JD-R-Modell sieht Arbeitsanforderungen („demands“) und Arbeitsressourcen („resources“) als unabhängige Faktoren, die Arbeitsbelastung und Arbeitsengagement beeinflussen. Hohe kognitive, emotionale und physische Anforderungen führen zu hoher Belastung, was langfristig negative Konsequenzen hat (u. a. Burn-out der Mitarbeitenden). Nach dem JD-R-Modell sind Arbeitsressourcen physische, soziale oder organisationale Aspekte der Arbeit, die helfen, Arbeitsanforderungen zu bewältigen. Dazu gehören u. a. kollegiale Unterstützung, Feedback, Möglichkeit zum Lernen und Bedeutung der Arbeit. Diese wirken entlastend, da sie menschliche Bedürfnisse nach Bindung, Selbstwertstärkung, Sicherheit und Kontrolle erfüllen. Vielfältige Arbeitsressourcen führen daher zu mehr Arbeitsengagement, was positive Konsequenzen für die Mitarbeitenden und das Unternehmen hat, z. B. in Form hoher Arbeitszufriedenheit.

**Abb. 1:**
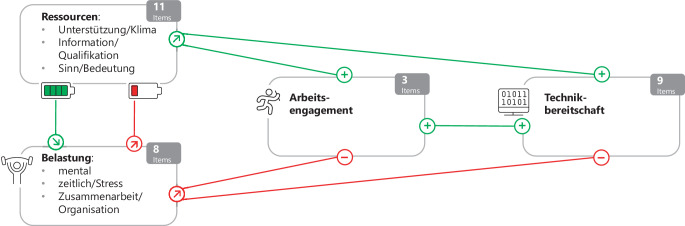
Zusammenfassung der arbeitsbezogenen Hypothesen im EXPERT-Projekt (Erläuterung im Text)

Das JDR-Modell geht davon aus, dass sich Arbeitsanforderungen und Arbeitsressourcen gegenseitig beeinflussen. Bei hohen Arbeitsanforderungen nehmen Mitarbeitende vorhandene Ressourcen vermehrt als nützlich wahr, was ihnen hilft, die Anforderungen zu bewältigen. Dies führt zu einer subjektiv geringeren Belastung [2]. In Abb. 1 sind die arbeitsbezogenen Hypothesen zusammengefasst: Hohe Arbeitsressourcen helfen, Arbeitsbelastungen zu bewältigen, hohe Belastungen verbrauchen vorhandene Ressourcen; hohe Arbeitsressourcen erhöhen das Arbeitsengagement und verbessern die Bereitschaft, neue Technologien zu nutzen; hohe Arbeitsbelastung hingegen verringert Engagement und Technikbereitschaft.

## Methoden

### Studiendesign

Für die medizinische und gesundheitsökonomische Evaluation des EXPERT-Projekts wurde ein Stepped-Wedge-Design gewählt, bei dem verschiedene Cluster zeitgleich mit einer Kontrollphase starten und dann kohortenweise in eine Interventionsphase übergehen [3, 4]. Die arbeitsbezogene Evaluation setzt auf diesem Design auf und nutzt den „Difference-in-Difference“-Ansatz [1, 11]: Kliniken, die bereits Empfehlungen des Boards erhalten (Treatment-Gruppe), werden mit Kliniken ohne Empfehlungen (Kontrollgruppe) verglichen. Die Mitarbeitenden bearbeiten an 2 Messzeitpunkten (prä vs. post) einen Online-Fragebogen zu ihrer Arbeitssituation, den sie über einen QR-Code auf ihrem Mobiltelefon aufrufen können. Zwischen den beiden Messzeitpunkten liegen 5 bis 6 Monate. Kurz nach der ersten Befragung (prä) wechselt die eine Hälfte der Kliniken in die Interventionsphase, während sich die andere Hälfte der Kliniken an beiden Messzeitpunkten in der Kontrollphase befindet. In allen Kliniken werden sowohl Personen aus der Ärzteschaft als auch aus nichtärztlichen Berufen, die direkt in EXPERT einbezogen sind, befragt. Die Teilnahme ist freiwillig und anonym. Die Auswertung erfolgt zusammenfassend über alle Kliniken. Im Folgenden wird über die Ergebnisse der Online-Befragung vor Beginn der Therapieempfehlungen berichtet.

#### Messinstrumente zur Befragung der Mitarbeitenden

Es wurden Messinstrumente eingesetzt, die *Arbeitsbelastung, Arbeitsengagement, arbeitsbezogene Ressourcen* und *Technikbereitschaft* erfassen, und für die empirische Vergleichswerte vorliegen. Alle Items wurden in einem EXPERT-Fragebogen zusammengestellt.

*Arbeitsbelastung *wurde anhand des NASA-Task Load Index in einer Version für Teamarbeit gemessen [5]. Es handelte sich um eine gekürzte Version mit 8 Items (Skalierung von 1 = sehr niedrig bis 10 = sehr hoch). Die Mitarbeitenden schätzten für die zurückliegenden 3 Monate die mentalen, zeitlichen und leistungsbezogenen Arbeitsanforderungen sowie den Aufwand für Austausch, Organisation der Arbeit, gegenseitige Unterstützung und Klären von Konflikten ein und gaben eine Einschätzung zu ihrer Zufriedenheit mit der Zusammenarbeit ab. Beispielweise lautete eine Formulierung: „Wie hoch war der Aufwand, die Arbeit zu organisieren (z. B. Abstimmung, wer welche Aufgabe übernimmt, wann welche Aufgabe erledigt ist)?“

*Arbeitsengagement *wurde mit der Ultra-Kurzform der Utrecht Work Engagement Scale [23] erhoben, die 3 Items enthält: „Bei der Arbeit bin ich voller Energie“, „Ich bin begeistert von meiner Arbeit“ und „Ich gehe ganz in meiner Arbeit auf“. Die internationale Form nutzt eine Skalierung von 0 = „nie“ bis 6 = „immer“, die hier verwendete deutsche Form eine entsprechende Skalierung von 1 bis 7 [28].

*Arbeitsressourcen* wurden mit dem Fragebogen zu Ressourcen und Anforderungen in der Arbeitswelt erfasst [23]. Es wurden 11 Items verwendet, die sich auf folgende Ressourcen beziehen: Arbeitsklima, Lernpotenzial, Bedeutung für Behandlung, Wissensweitergabe, vorbereitende Information, Unterstützung durch Führungskraft, kollegiale Unterstützung, Feedback zur Arbeit, abwechslungsreiche Aufgaben, klare Zuständigkeiten und Qualifikation (Tab. 2). Alle Items wurden als Aussagen formuliert, inhaltlich bezogen auf das EXPERT-Projekt (Skalierung von 1 = trifft gar nicht zu bis 6 = trifft vollkommen zu), wie z. B.: „Ich kann mich bei der Vorbereitung auf die neuen Maßnahmen auf die Unterstützung meiner Führungskraft verlassen.“

Die *Bereitschaft, neue Technologien zu nutzen,* wurde mit der Skala zur Erfassung von Technikbereitschaft (TB) gemessen [18], die 9 Items in 3 Skalen „TB-Akzeptanz“, „TB-Kontrollüberzeugung“ und „TB-Kompetenz“ enthält (Skalierung 1 = trifft gar nicht zu bis 5 = trifft völlig zu). Die Item-Formulierungen wurden an das EXPERT-Projekt angepasst, z. B.: „An der neuen telemedizinischen Anwendung bin ich sehr interessiert“ (TB-Akzeptanz), „Es hängt im Wesentlichen von mir ab, ob ich im Einsatz der neuen telemedizinischen Anwendung erfolgreich bin“ (TB-Kontrollüberzeugung) und „Für mich stellt der Umgang mit technischen Neuerungen meistens eine Überforderung dar“ (TB-Kompetenz).

Abschließend gaben die Befragten Auskunft zu ihrem Beruf (ärztlich/nichtärztlich), ob sie eine Führungsposition ausübten (Ja/Nein) und ob sie bereits vor dem EXPERT-Projekt über Erfahrungen in der Anwendung von Telemedizin verfügten (Ja/Nein).

#### Statistische Auswertung

Ausgewertet wurden der Einfluss der Faktoren Berufsgruppe, Leitungsfunktion, Arbeitsbelastung individuell und teambezogen (hoch vs. niedrig) und Arbeitsressourcen (hoch vs. niedrig) auf das Arbeitsengagement und die Technikbereitschaft. Für die beiden zuletzt genannten Faktoren wurde jeweils der Median bestimmt. Alle Werte unterhalb des Medians wurden als „niedrig“, über dem Median als „hoch“ gewertet. Es wurde eine multiple Varianzanalyse (MANOVA) zum Einfluss der Gruppierungsfaktoren Individualbelastung (hoch vs. niedrig), teambezogene Belastung (hoch vs. niedrig) und Arbeitsressourcen (hoch vs. niedrig) auf Arbeitsengagement und Technikbereitschaft mit den Kovariaten Berufsgruppe und Leitungsfunktion gerechnet. Zudem wurde eine kanonische Diskriminanzanalyse zum Einfluss der verschiedenen arbeitsbezogenen Ressourcen auf die Höhe des Arbeitsengagements und der Technikbereitschaft durchgeführt.

## Ergebnisse

### Stichprobe

Insgesamt nahmen 84 Personen aus 22 Kliniken an der Befragung zum Zeitpunkt prä teil, davon 46 Personen des ärztlichen und 36 Personen des nichtärztlichen Personals (Pflegekräfte, medizinische Fachangestellte bzw. Physician Assistants). Davon waren 34 Personen in leitender, 44 Personen in nichtleitender Funktion. 6 Personen machten hierzu keine Angaben. Eine Übersicht über die Teilnahmezahlen gibt Tab. 1.

**Tab. 1:** Anzahl der Teilnehmenden zum Zeitpunkt prä, zusammengefasst nach Berufsgruppe und Leitungsfunktion

		Berufsgruppe			Gesamt
		Ärztliches Personal	Nichtärztlich (Pflege, MFA, PA)	Keine Angabe	
Sind Sie in leitender Funktion tätig?	Ja	27	7	–	34
	Nein	18	26	–	44
Keine Angabe		1	3	2	6
Gesamt		46	36	2	84

### Gesamtstichprobe: starkes Arbeitsengagement bei hoher Arbeitsbelastung

**Abb. 2:**
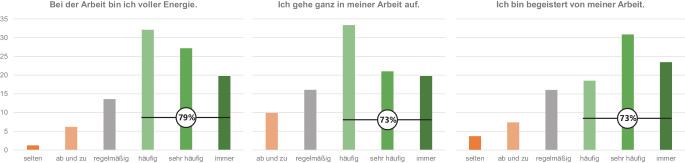
Häufigkeitsverteilungen der 3 Fragen zum Arbeitsengagement (von 1 = „nie“ bis 7 = „immer“; Hinweis: 1 = „nie“ kam nicht vor)

Das Arbeitsengagement vor Beginn der EXPERT-Maßnahmen war sehr hoch. 79 % der Befragten gaben an, bei der Arbeit „voller Energie“ zu sein, 73 % sagten, „ganz in ihrer Arbeit aufzugehen“, und 73 % zeigten sich „begeistert“ von ihrer Arbeit (Abb. 2). Mit einem Durchschnittswert von 5,3 lagen die Befragten über dem Vergleichswert von 4,9 für eine deutsche Stichprobe mit 1406 Befragten [28], im Bereich des Mittelwerts von 5,1 für 223 deutsche Ärztinnen und Ärzte [14] und beim Höchstwert von 5,3 in einer internationalen Vergleichsstudie mit über 77.000 Teilnehmenden [23].

Von den Befragten schätzten 86 % ihre individuelle Arbeitsbelastung als hoch bis sehr hoch ein. Die Zusammenarbeit im Team verlief überwiegend gut: Nur 37 % der Befragten gaben eine hohe Belastung in der Zusammenarbeit im Team an. Es wurden keine Unterschiede zwischen den Berufsgruppen gefunden. Die Gesamtstichprobe wies eine durchschnittliche Bereitschaft, die für das Projekt neu entwickelte Software zu nutzen, auf.

### Haupteffekte und Wechselwirkungen

#### Zusammenhänge von Arbeitsengagement, Arbeitsressourcen und Technikbereitschaft mit Berufsgruppen

Die beiden Berufsgruppen unterschieden sich weder hinsichtlich ihres Arbeitsengagements (5,44 vs. 5,20, n. s.) noch ihrer Technikbereitschaft (3,86 vs. 3,96, n. s.).

**Abb. 3:**
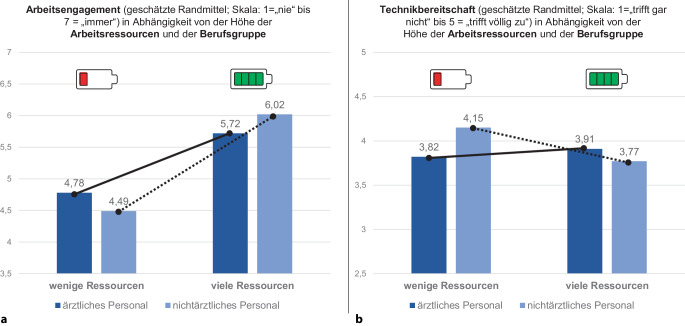
Effekt von Berufsgruppe (ärztlich vs. nichtärztlich) und arbeitsbezogenen Ressourcen (niedrig vs. hoch) auf Arbeitsengagement (**a**) und Technikbereitschaft (**b**)

Wie erwartet, war in beiden Berufsgruppen das Arbeitsengagement höher, wenn mehr Ressourcen zur Verfügung standen (niedrige Ressourcen = 4,53 vs. hohe Ressourcen = 5,95; F[1,67] = 8,3; *p* < 0,001; Abb. 3a, angegeben sind die korrigierten Randmittelwerte für beide Berufsgruppen).

Im Hinblick auf den Zusammenhang von Arbeitsressourcen und Technikbereitschaft gab es eine signifikante Wechselwirkung von Berufsgruppe und Ausmaß der Ressourcen: Während bei ärztlichem Personal die Technikbereitschaft bei mehr Arbeitsressourcen etwas höher war (niedrige Ressourcen = 3,82 vs. hohe Ressourcen = 3,91), war die Technikbereitschaft bei nichtärztlichem Personal höher, wenn weniger Arbeitsressourcen zur Verfügung standen (niedrige Ressourcen = 4,15 vs. hohe Ressourcen = 3,77; F[1,67] = 5,7; *p* < 0,05; Abb. 3b, angegeben sind die korrigierten Randmittelwerte für beide Berufsgruppen).

Eine Leitungsfunktion führte zu einer signifikant höheren Technikbereitschaft (4,13 vs. 3,73; F[1,67] = 4,4; *p* < 0,05), jedoch nicht zu signifikant erhöhtem Arbeitsengagement (5,35 vs. 5,13, n. s.).

Zur Verbesserung des Arbeitsengagements trugen alle arbeitsbezogenen Ressourcen bei, wie eine Diskriminanzanalyse zum Zusammenhang der Arbeitsressourcen [24] mit dem Arbeitsengagement [28] zeigt (Tab. 2; alle Ergebnisse *p* < 0,01; aufgeklärte Varianz korr. R^2^ 81 %). Die wichtigsten Ressourcen, in der Reihenfolge ihrer Bedeutung, waren: 1. positives Arbeitsklima, 2. Lernen zur Vorbereitung, 3. erkennbare Bedeutung der Arbeit für den Behandlungserfolg, 4. interne Wissensweitergabe, 5. rechtzeitige Information sowie 6. kollegiale Unterstützung und 7. Führungskräfte. Als weniger wichtig erwies sich eine bereits vorab bestehende Qualifikation für die neuen Aufgaben, weil eine passende Qualifizierung ja gerade im Laufe des Projekts erworben werden soll.

**Tab. 2:** Ergebnisse einer kanonischen Diskriminanzanalyse zur Auswirkung der Arbeitsressourcen auf die Höhe des Arbeitsengagements

Item Arbeitsressourcen	Fisher’s Z	Wilks-Lambda	F[3,76]	Sign.
*Arbeitsklima*: Unser Arbeitsklima bei der Vorbereitung auf die neuen Maßnahmen ist ausgezeichnet	0,71^*^	0,66	13,11	< 0,001
*Lernpotenzial*: Bei der Vorbereitung auf die neuen Maßnahmen lerne ich viel dazu	0,67^*^	0,68	12,21	< 0,001
*Bedeutung für Behandlung*: Meine Aufgaben bei der Vorbereitung auf die neuen Maßnahmen sind sehr wichtig für die Behandlung der Patientinnen und Patienten	0,62^*^	0,71	10,32	< 0,001
*Wissensweitergabe*: In meiner Klinik wird das Wissen über die Vorbereitung auf die neuen Maßnahmen weitergegeben	0,61^*^	0,72	9,99	< 0,001
*Vorbereitende Information*: Ich werde bei der Vorbereitung auf die neuen Maßnahmen über alle wichtigen Inhalte informiert	0,55^*^	0,75	8,42	< 0,001
*Kollegiale Unterstützung*: Ich kann mich bei der Vorbereitung auf die neuen Maßnahmen auf die Unterstützung durch meine Kolleginnen und Kollegen verlassen	0,53^*^	0,76	8,00	< 0,001
*Unterstützung durch Führungskraft*: Ich kann mich bei der Vorbereitung auf die neuen Maßnahmen auf die Unterstützung meiner Führungskraft verlassen	0,54^*^	0,76	7,86	< 0,001
*Feedback zur Arbeit*: Ich erhalte bei der Vorbereitung auf die neuen Maßnahmen sehr hilfreiches Feedback zu meiner Arbeit	0,41	0,82	5,70	< 0,01
*Abwechslungsreiche Aufgaben*: Meine Aufgaben bei der Vorbereitung auf die neuen Maßnahmen sind sehr abwechslungsreich	0,41	0,82	5,65	< 0,01
*Klare Zuständigkeiten*: Bei der Vorbereitung auf die neuen Maßnahmen weiß ich immer, wer wofür zuständig ist	0,46	0,80	6,42	< 0,01
*Qualifikation*: Ich habe die nötige Qualifikation für die neuen Aufgaben	0,29	0,90	2,92	< 0,05

#### Einfluss von individueller und teambezogener Arbeitsbelastung sowie Arbeitsressourcen auf Engagement und Technikbereitschaft

**Abb. 4:**
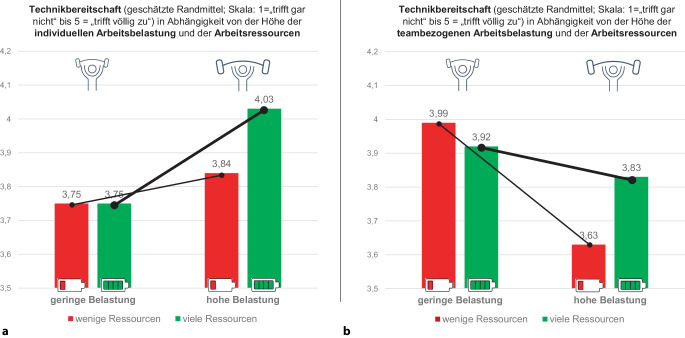
Höhe der Technikbereitschaft in Abhängigkeit von der individuellen Belastung (**a**) und teambezogenen Belastung (**b**) sowie der arbeitsbezogenen Ressourcen

Weder die individuelle noch die teambezogene Arbeitsbelastung allein hatten signifikante Auswirkungen auf das Arbeitsengagement und die Technikbereitschaft. Stattdessen ergaben sich signifikante Wechselwirkungen: Bei geringer Individualbelastung und wenigen Arbeitsressourcen war die Technikbereitschaft niedrig. Bei hoher Individualbelastung stieg die Technikbereitschaft deutlich an, wenn viele arbeitsbezogene Ressourcen verfügbar waren (F[1,67] = 5,9; *p* < 0,01; Abb. 4a).

Anders sah es bei teambezogener Belastung aus: Eine geringe Belastung im Rahmen der Teamarbeit führte zu hoher Technikbereitschaft, unabhängig von dem Ausmaß der Arbeitsressourcen. Hingegen war bei hoher teambezogener Belastung, z. B. durch Konflikte, das Ausmaß der Arbeitsressourcen entscheidend für die Techniknutzung: Bei hoher teambezogener Belastung und nur wenigen Ressourcen war die Technikbereitschaft besonders gering (F[1,67] = 5,4; *p* < 0,05; Abb. 4b).

Sowohl bei hoher individueller als auch bei hoher teambezogener Arbeitsbelastung waren Arbeitsressourcen also entscheidend für die Bereitschaft, neue Technologien zu nutzen. Allerdings war die Wirkungsweise verschieden: Viele Arbeitsressourcen führten bei hoher individueller Belastung zu höherer Technikbereitschaft. Ein Mangel an Arbeitsressourcen hingegen führte bei hoher teambezogener Belastung zu geringer Technikbereitschaft. Daraus ergab sich eine signifikante dreifache Wechselwirkung (Abb. 4a vs. **b**; F[1,67] = 4,8; *p* < 0,05).

## Diskussion

Das Arbeitsengagement in den 22 erfassten Kliniken ist sehr hoch. Das ist ein starkes Zeichen für einen hohen persönlichen Einsatz im Heilberuf. Dies ist umso bemerkenswerter, da die individuelle Arbeitsbelastung ebenfalls als hoch bewertet wird. Die Teamarbeit verläuft weitgehend unkompliziert. Es wurde eine enge Wechselwirkung zwischen Arbeitsressourcen, Arbeitsbelastung und Technikbereitschaft festgestellt. Während eine hohe individuelle Arbeitsbelastung durch ausreichende Arbeitsressourcen kompensiert werden konnte und die Technikbereitschaft steigerte, führte eine hohe teambezogene Belastung bei gleichzeitig geringen Ressourcen zu einer deutlichen Verringerung der Technikakzeptanz. Besonders auffällig war, dass nichtärztliches Personal neue Technologien tendenziell eher als Unterstützung wahrnahm, insbesondere dann, wenn wenige Arbeitsressourcen verfügbar waren.

## Schlussfolgerungen

Die Befunde bestätigen zentrale Annahmen des Job-Demands-Resources-Modells: Arbeitsressourcen spielen eine entscheidende Rolle für das Arbeitsengagement und die Bereitschaft zur Nutzung neuer Technologien. Hohe individuelle Belastungen können durch geeignete Ressourcen, insbesondere positives Arbeitsklima, Lernmöglichkeiten und Bedeutung der Arbeit für die Patientenversorgung, abgefedert werden, sodass die Nutzung neuer Technologien als entlastende Maßnahme empfunden wird. Hingegen wird bei hoher teambezogener Belastung der Fokus der verfügbaren Ressourcen auf die direkte Teamarbeit gelenkt, was zu einer geringeren Technikbereitschaft führt. Die Studie deutet darauf hin, dass nichtärztliches Personal unter Bedingungen begrenzter Ressourcen neue Technologien stärker als Entlastung wahrnimmt als ärztliches Personal.

### Limitationen

Da sich von den 31 Kliniken, die in das EXPERT-Projekt einbezogen sind, nur 22 Kliniken an der Befragung zum Zeitpunkt prä beteiligt haben, kann ein Selektionseffekt im Hinblick auf die Stichprobe nicht ausgeschlossen werden. Die Erhebung wurde zu einem frühen Zeitpunkt der Implementierung des telemedizinischen Extremitäten-Boards durchgeführt, daher können noch keine Aussagen über langfristige Effekte getroffen werden. Die Technikbereitschaft beruht auf einer Selbsteinschätzung der Befragten, ohne direkte Messung der tatsächlichen Techniknutzung.

## Fazit für die Praxis

- Vielfältige arbeitsbezogene Ressourcen stärken das Arbeitsengagement und die Akzeptanz digitaler Innovationen.
- Wichtig sind dabei die gezielte Unterstützung interdisziplinärer Teams durch verbesserte Arbeitsorganisation und die Förderung einer positiven Teamkultur.
- Die Ergebnisse zeigen, dass man sich auf das Berufsethos der im Krankenhaus Beschäftigten verlassen kann. Das darf aber nicht darüber hinwegtäuschen, dass die Arbeitsbelastung hoch ist, und insbesondere schlecht geführte, belastete Teams sehr viele Arbeitsressourcen „verbrauchen“, sodass für die Nutzung neuer Technik kaum noch Kraft bleibt.
- Damit das hohe Arbeitsengagement langfristig erhalten bleibt, sind ein positives Arbeitsklima, verbunden mit einer Kultur der kollegialen Unterstützung, Lernmöglichkeiten und die erkennbare Bedeutung der Arbeit für die Patientenversorgung entscheidend. Die Einführung neuer Technologien sollte strategisch begleitet werden, insbesondere in hochbelasteten Teams, um Akzeptanz zu fördern. Durch eine gezielte Fokussierung auf psychologische, soziale und organisationale Arbeitsressourcen kann durch höhere Arbeitszufriedenheit auch die Patientenversorgung gestärkt werden.

## Data Availability

The raw data have been made available (format SPSS file sav) under 10.13140/RG.2.2.29741.19689.

## References

[1] Abadie A (2005) Semiparametric difference-in-differences estimators. Rev Econ Stud 72(1):1–9

[2] Bakker AB, Demerouti E, Sanz-Vergel AI (2014) Burnout and work engagement: The JD‑R approach. Annu Rev Organ Psychol Organ Behav 1(1):389–411

[3] Binik A Delaying and withholding interventions: ethics and the stepped wedge trial. J Med Ethics 45(10):662–667 10.1136/medethics-2018-105138 31341013

[4] Brown CA, Lilford RJ (2006) The stepped wedge trial design: a systematic review. BMC Med Res Methodol 6:54 17092344 10.1186/1471-2288-6-54 PMC1636652

[5] Coelho DA, Filipe JN, Simoes-Marques M, Nunes IL (2014) The expanded cognitive task load index (NASA-TLX) applied to team decision-making in emergency preparedness simulation. In: Proceedings of the 2014 Annual Conference on Human Factors and Ergonomics Society Europe Annual Conference. https://www.hfes-europe.org/largefiles/proceedingshfeseurope2014.pdf (Presented at: HFES ’14; October 8–10, 2014:8–10; Lisbon, Portugal)

[6] Demerouti E, Bakker A, Nachreiner F, Schaufeli WB (2000) A model of burnout and life satisfaction amongst nurses. J Adv Nurs 32(2):454–464 10964195 10.1046/j.1365-2648.2000.01496.x

[7] Demerouti E, Nachreiner F (2019) Zum Arbeitsanforderungen-Arbeitsressourcen-Modell von Burnout und Arbeitsengagement – Stand der Forschung. Z Arb Wiss 73(2):119–130

[8] Elniel AR, Giannoudis PV (2018) Open fractures of the lower extremity: Current management and clinical outcomes. Efort Open Rev 3(5):316–325 29951271 10.1302/2058-5241.3.170072 PMC5994617

[9] Giannoudis PV, Harwood PJ, Kontakis G, Allami M, Macdonald D, Kay SP, Kind P (2009) Long-term quality of life in trauma patients following the full spectrum of tibial injury (fasciotomy, closed fracture, grade IIIB/IIIC open fracture and amputation). Injury 40(2):213–219 19070847 10.1016/j.injury.2008.05.024

[10] Goff DA, Kullar R, Goldstein EJ, Gilchrist M, Nathwani D, Cheng AC, Cairns KA, Escandón-Vargas K, Villegas MV, Brink A, van den Bergh D (2017) A global call from five countries to collaborate in antibiotic stewardship: united we succeed, divided we might fail. Lancet Infect Dis 2017 Feb 1 17(2):e56–e63 10.1016/S1473-3099(16)30386-3 27866945

[11] Graves JA, Fry C, McWilliams JM, Hatfield LA (2022) Difference-in-differences for categorical outcomes. Health Serv Res 57(3):681–692 35132619 10.1111/1475-6773.13948 PMC9108044

[12] Hoekstra H, Smeets B, Metsemakers WJ, Spitz AC, Nijs S (2017) Economics of open tibial fractures: the pivotal role of length-of-stay and infection. Health Econ Rev 7(1):32 28948497 10.1186/s13561-017-0168-0 PMC5612906

[13] Karanika S, Paudel S, Grigoras C, Kalbasi A, Mylonakis E (2016) Systematic review and meta-analysis of clinical and economic outcomes from the implementation of hospital-based antimicrobial stewardship programs. Antimicrob Agents Chemother 60(8):4840–4852 27246783 10.1128/AAC.00825-16 PMC4958232

[14] Mache S, Vitzthum K, Wanke E, David AFKB, Klapp BF, Danzer G (2014) Exploring the impact of resilience, self-efficacy, optimism and organizational resources on work engagement. Work 47(4):491–500 23531578 10.3233/WOR-131617

[15] Metsemakers WJ, Morgenstern M, Senneville E, Borens O, Govaert GA, Onsea J, Depypere M, Richards RG, Trampuz A, Verhofstad MH, Kates SL (2020) General treatment principles for fracture-related infection: recommendations from an internation al expert group. Arch Orthop Trauma Surg 140(8):1013–1027 31659475 10.1007/s00402-019-03287-4 PMC7351827

[16] Murdoch DR, Roberts SA, Fowler VG Jr, Shah MA, Taylor SL, Morris AJ, Corey GR (2001) Infection of orthopedic prostheses after Staphylococcus aureus bacteremia. Clin Infect Dis 32(4):647–649 11181131 10.1086/318704

[17] Nanchahal J, Nayagam S, Kahn U, Moran C, Barrett S, Sanderson F et al (2009) Standards for the Management of Open Fractures of the Lower Limb. Marston Book, Abingdon, UK

[18] Neyer FJ, Felber J, Gebhardt C (2012) Entwicklung und Validierung einer Kurzskala zur Erfassung von Technikbereitschaft. Diagnostica 58(2):87–99

[19] Oflazoglu K, Hoogendoorn JM, van der Zwaal P, Walbeehm ET, van Enst WA, Holtslag HR, Hofstee D, Plantinga P, Elzinga M, Rakhorst H (2019) Treating open lower limb fractures successfully; thoughts and current practice on therapy and centralization in The Netherlands. Eur J Trauma Emerg Surg 45(1):99–106 29181549 10.1007/s00068-017-0874-7 PMC6394538

[20] Rees S, Tutton E, Achten J, Bruce J, Costa ML (2019) Patient experience of long-term recovery after open fracture of the lower limb: a qualitative study using interviews in a community setting. Bmj Open 9(10):e31261 31601595 10.1136/bmjopen-2019-031261 PMC6797425

[21] Rodriguez L, Jung HS, Goulet JA, Cicalo A, Machado-Aranda DA, Napolitano LM (2014) Evidence-based protocol for prophylactic antibiotics in open fractures: improved antibiotic stewardship with no increase in infection rates. J Trauma Acute Care Surg 77(3):400–408 25159242 10.1097/TA.0000000000000398

[22] Rosslenbroich S, Laumann M, Hasebrook J, Rodde S, Grosser J, Greiner W, Hirsch T, Windrich S, Raschke MJ (2024) Improving the Care of Severe, Open Fractures and Postoperative Infections of the Lower Extremities: Protocol for an Interdisciplinary Treatment Approach. JMIR Res Protoc 13:e57820 39284180 10.2196/57820 PMC11451582

[23] Schaufeli WB, Shimazu A, Hakanen J, Salanova M, De Witte H (2019) An ultra-short measure for work engagement: the UWES‑3 validation across five countries. Eur J Psychol Assess 35(4):577–591

[24] Schulte EM, Wittner B, Kauffeld S (2021) Ressourcen und Anforderungen (ReA) in der Arbeitswelt: Entwicklung und erste Validierung eines Fragebogens. Gr Interakt Org 52(2):405–415

[25] Tampe U, Weiss RJ, Stark B, Sommar P, Al Dabbagh Z, Jansson KÅ (2014) Lower extremity soft tissue reconstruction and amputation rates in patients with open tibial fractures in Sweden during 1998–2010. BMC Surg 14:80 25323662 10.1186/1471-2482-14-80 PMC4202253

[26] Trampuz A, Zimmerli W (2006) Diagnosis and treatment of infections associated with fracture-fixation devices. Injury 37(2):59–66 10.1016/j.injury.2006.04.010 16651073

[27] Turrentine FE, Henderson WG, Khuri SF, Schifftner TL, Inabnet WB, El-Tamer M et al (2007) Adrenalectomy in veterans affairs and selected university medical centers: results of the patient safety in surgery study. J Am Coll Surg 204(6):1273–1283 17544085 10.1016/j.jamcollsurg.2007.03.014

[28] Ulusoy N, Mölders C, Fischer S, Bayur H, Deveci S, Demiral Y, Rössler W (2016) Utrecht Work Engagement Scale—German Version. APA PsycTests. 10.1037/t53265-000

